# Supplementary figures and images for: Heading for Personalized rTMS in Tinnitus: Reliability of Individualized Stimulation Protocols in Behavioral and Electrophysiological Responses
Source: J Pers Med. 2021 Jun 9;11(6):536. doi: 10.3390/jpm11060536 (PMC8226921; doi:10.3390/jpm11060536)

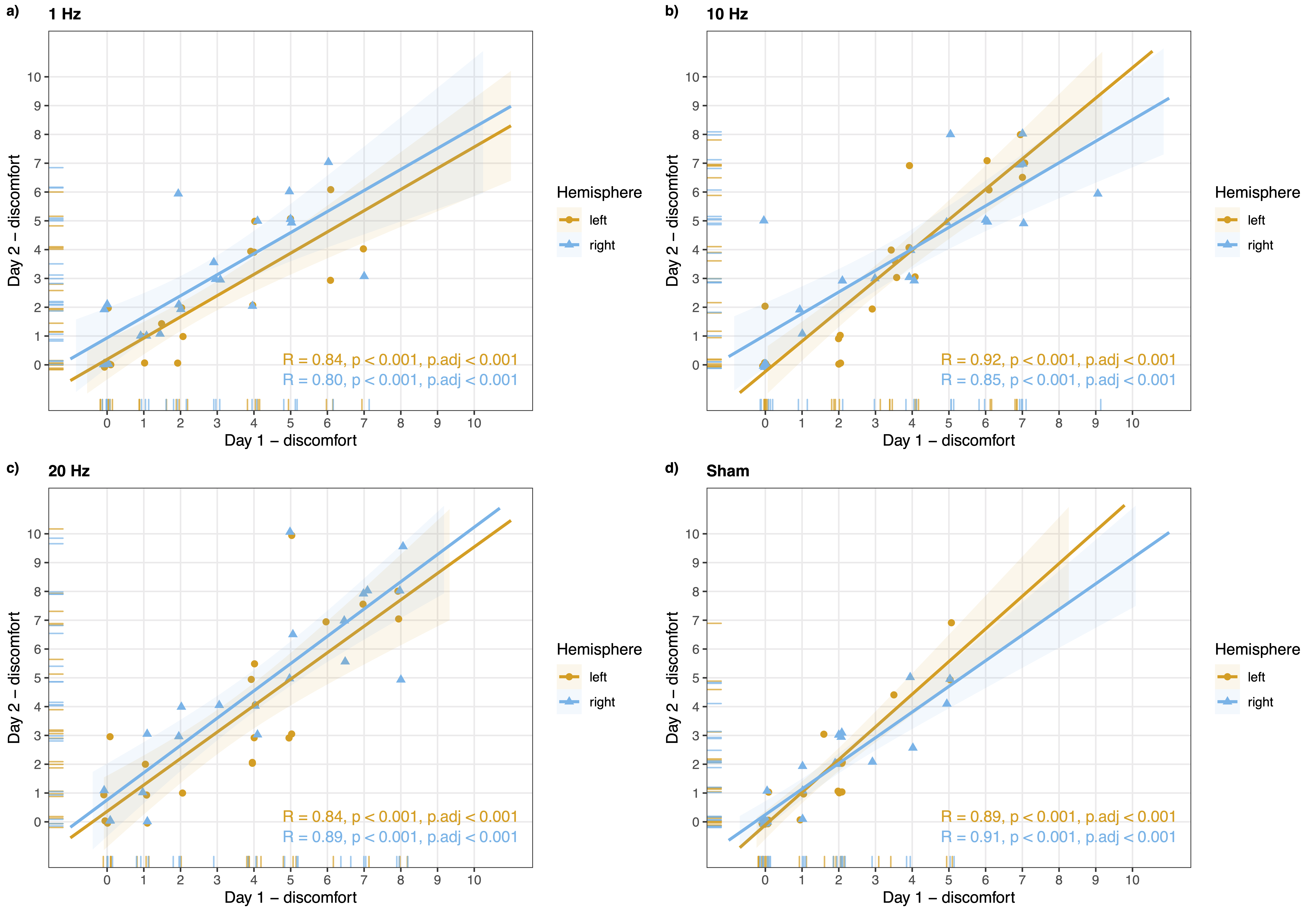

Supplement: Supplementary file 1 [file jpm-11-00536-s001.zip › Supplemental_Material/FigureS1_discomfort.png]
